# Beyond perceptual load and dilution: a review of the role of working memory in selective attention

**DOI:** 10.3389/fpsyg.2013.00287

**Published:** 2013-05-21

**Authors:** Jan W. de Fockert

**Affiliations:** Department of Psychology, Goldsmiths, University of LondonLondon, UK

**Keywords:** selective attention, distractibility, working memory load, working memory capacity, review, meta-analysis

## Abstract

The perceptual load and dilution models differ fundamentally in terms of the proposed mechanism underlying variation in distractibility during different perceptual conditions. However, both models predict that distracting information can be processed beyond perceptual processing under certain conditions, a prediction that is well-supported by the literature. Load theory proposes that in such cases, where perceptual task aspects do not allow for sufficient attentional selectivity, the maintenance of task-relevant processing depends on cognitive control mechanisms, including working memory. The key prediction is that working memory plays a role in keeping clear processing priorities in the face of potential distraction, and the evidence reviewed and evaluated in a meta-analysis here supports this claim, by showing that the processing of distracting information tends to be enhanced when load on a concurrent task of working memory is high. Low working memory capacity is similarly associated with greater distractor processing in selective attention, again suggesting that the unavailability of working memory during selective attention leads to an increase in distractibility. Together, these findings suggest that selective attention against distractors that are processed beyond perception depends on the availability of working memory. Possible mechanisms for the effects of working memory on selective attention are discussed.

## Introduction

The question of when visual selection takes place during information processing has been a major issue in selective attention research that long remained unresolved, given evidence for both early and late selection. Early selection is suggested by the finding, among others, of no evidence for identification of unattended information (e.g., Lachter et al., 2004). Conversely, peripheral irrelevant distractor letters tend to interfere with the identification of central target letters, suggesting that the distractors were processed at least to the level of letter identity (e.g., Eriksen and Eriksen, 1974). This implies late selection. Perceptual load theory (Lavie and Tsal, 1994; Lavie, 1995) offered a resolution to the debate, by suggesting that the locus of selection was dependent on the perceptual processing demands of the task at hand: high perceptual demands would prevent any distracting information to be processed, leading to early selection, whereas low perceptual demands would allow for the processing of distractors, necessitating late selection.

Studies directly investigating the effect of perceptual load long seemed to support the idea that the relevant perceptual processing demands determine the extent of processing for irrelevant information (e.g., Lavie, 1995; Lavie and Cox, 1997; Lavie et al., 2004). Recently, however, a rival explanation has been put forward, suggesting that the reduction in distractibility under high perceptual load is due to greater dilution of the distractor (Benoni and Tsal, 2010, 2012; Tsal and Benoni, 2010; Wilson et al., 2011). On this view, the high perceptual load conditions are associated with reduced distractibility simply because the distractors compete with the additional relevant non-targets in high load displays, rather than exhaustion of attentional capacity under high perceptual load.

The main difference between the load and dilution models concerns the mechanisms underlying perceptual selectivity, whereas both models assume that distractors are more likely to be processed under certain perceptual conditions. In such cases of relatively extensive distractor processing, behavior nonetheless remains largely goal-appropriate. In other words, although the processing of the perceived distractors under low perceptual load or low dilution has a measurable effect on target processing, observers are still able to prioritize target processing, and prevent for example responding inappropriately to distracting information. A key question therefore is: how are processing priorities maintained in order to achieve target-directed behavior when current perceptual aspects do not allow for sufficient attentional selectivity and distractors are likely to receive considerable processing?

Dilution involves a process of early selection: whether or not a distractor receives processing depends on perceptual characteristics of the visual display. As such, the dilution model makes no specific predictions regarding the fate of distracting information that has not been excluded from processing by dilution. Tsal and Benoni (2010) do offer an interpretation of load theory, suggesting that any additional increase in the load on attentional resources should reduce the likelihood that attentional resources spill over to the distractors. On this view, an increase in attentional load, even when it does not affect perceptual task aspects, should be associated with reduced distractor processing. As we outline below, this is not the case, and increases in attentional load that involve top-down attentional control, rather than stimulus-determined selection processes such as perceptual load, tend to have the effect of enhancing distractor processing.

Load theory suggests that cognitive control functions supported by the frontal lobes, particularly working memory, are critical in late selection (Lavie et al., 2004; Lavie, 2010). On this view, working memory plays a key role in maintaining processing priorities, so that target and distractor-related information remains clearly separated in processing, and behavior can be successfully directed toward task-relevant information. This idea was not new, and an earlier suggestion that working memory and selective attention may show functional overlap came in the context of Baddeley's working memory model (Baddeley, 1996), which argued that a key function of the central executive component of working memory is to facilitate selective attention to relevant information in the presence of potential distractions. At the time, the evidence for an association between working memory and selective attention was indirect and came from two lines of investigation. First, studies on age-related changes in selective attention had shown that the ability to prevent distraction by irrelevant information is disproportionately affected by age (e.g., Rabbitt, 1965; Hasher and Zacks, 1988). Together with the finding that working memory performance also deteriorates with age (e.g., Welford, 1958; Morris et al., 1988), this provided indirect evidence that working memory may somehow be involved in achieving selective processing. A second early suggestion that working memory may be involved in selective attention was made by Desimone and Duncan (1995), who argued that control of attention from the top down (i.e., not entirely based on attention-grabbing properties of the input), involves maintenance of a template specifying what information is relevant for the task at hand, a function ideally suited to working memory.

The first study to provide direct evidence for a role of working memory in selective attention used a paradigm combining a working memory task with a selective attention task to measure distractor interference in a context of varying working memory load (De Fockert et al., 2001). In that study, people performed a target name classification task (popstar, politician) while ignoring distractor faces (Young et al., 1986), so that any processing of the irrelevant faces would lead to poorer performance on trials on which the face category was incompatible with the current target name category (e.g., the name Elton John accompanied by the face of Bill Clinton), compared with trials on which the name and face categories were either compatible (e.g., the name and face of Elton John) or unrelated (e.g., the name Elton John with an anonymous face). The selective attention task was performed in a context of either low or high working memory load. At the start of each trial, people saw a set of five digits that they had to remember until the end of the trial in order to be able to respond to a memory probe. Working memory load was manipulated by varying the order of the set digits: on low load trials, digits always appeared in a sequential order, whereas a different random order was used on high load trials. The prediction was that, if working memory is important for maintaining selective attention to the relevant target names, then making it relatively unavailable to do so (by involving working memory in an additional task of high load) should lead to less selective processing. The results supported this prediction. Compatibility effects in terms of reaction times and accuracy rates were greater under high working memory load (78 ms difference between compatible and incompatible displays), compared to low load (46 ms difference). Moreover, the neural response in brain areas dedicated to processing the irrelevant faces was also greater under high (vs. low) working memory load. Following the initial indirect suggestions for a role of working memory in selective attention (Desimone and Duncan, 1995; Baddeley, 1996), this provided the first strong evidence that within the same participants, and without changing any properties of the selective attention task, working memory can be shown to affect visual distractibility.

The initial demonstration of a role of working memory in selective attention has prompted much further work on the association between these constructs. The main aim of this paper is to provide an overview of the evidence so far, focusing on the question of how the availability of working memory affects distractibility in selective attention. Other important aspects regarding the relation between working memory and selective attention, including how attention determines which information is entered into, and prioritized within, working memory (e.g., Oberauer, 2003; Gazzaley and Nobre, 2012), and how attention can be biased toward the contents of working memory (Awh and Jonides, 2001; Soto et al., 2008; Olivers et al., 2011), are not covered here. Instead, we will focus specifically on studies that manipulate the level of working memory availability during attention, and measure distractibility as a function of either load on working memory, or working memory capacity. As we will see, the majority of studies indeed show that the unavailability of working memory for selective attention leads to an increase in distractibility, although we will also discuss evidence suggesting that loading working memory can result in a reduction in distractibility in certain circumstances. Finally, studies elucidating the mechanism of how working memory may affect selective attention are discussed.

## Working memory load and distractibility

Since the first experimental report that high working memory load can lead to greater processing of irrelevant information, a number of studies have found similar effects. The load-related increase in the interference effect from irrelevant faces in the popstar/politician task has been replicated in two experiments (Pecchinenda and Heil, 2007, Experiments 1 and 2; but see Jongen and Jonkman, 2011, for an example in which working memory load increased face distraction, but not significantly so). In a standard Eriksen-type flanker task (Eriksen and Eriksen, 1974), in which attention needs to be selectively directed toward a target letter in the presence of a distracting flanker letter, the interference effect produced by the flanker (measured by comparing performance to displays in which the distractor is compatible vs. incompatible with the target) is significantly enhanced during concurrent retention of a memory set of six digits (vs. one digit) (Lavie et al., 2004, Experiment 1; De Fockert et al., 2010; Ahmed and De Fockert, 2012b). Adding a working memory task has a similar effect of increasing the processing of irrelevant flankers other than letters, such as left or right-pointing arrows (Pratt et al., 2011). Both the popstar/politician task and the flanker tasks produce Stroop-type interference effects, whereby to-be-ignored distractors (faces, flanker letters, or arrows) are task-relevant to the extent that they are associated with a possible response. Indeed, color word interference in a classic Stroop task is also greater when load on working memory is high (Stins et al., 2004). The distractors appear on every trial in these tasks, making their presence perfectly predictable. That high working memory load increases the extent to which such distractors are processed, suggests that working memory is involved in the active suppression of known distractors.

A similar effect of working memory load of increasing distractor processing has been shown for distractors that are not task-relevant, and whose presence is not perfectly predictable. In visual search tasks, the presence of a salient singleton distractor leads to a performance impairment that is usually interpreted to reflect the capture of attention by the distractor (see Theeuwes, 2010, for a review). For example, search for a shape target is slower when one of the non-target display items has a unique color, even though color is an irrelevant dimension throughout the experiment (Theeuwes, 1992). Such attentional capture effects are greater under high working memory load (Boot et al., 2005; Lavie and De Fockert, 2005, 2006, but see De Fockert and Theeuwes, 2012, for an example where high working memory load did not modulate capture effects), suggesting that working memory is involved in minimizing the distraction caused by the singleton colors. Indirectly, the interpretation that singleton capture involves cognitive control, possibly working memory, is further supported by neuroimaging findings showing that the magnitude of capture in behavior is negatively correlated with activity in left frontal cortex (De Fockert et al., 2004). In addition to actively minimizing the capturing effect of the singletons, working memory may also be involved in merely detecting their presence. Activity in right prefrontal cortex is correlated with the magnitude of behavioral capture, this time showing a positive association, and only when concurrent working memory load is high (De Fockert and Theeuwes, 2012). This suggests that working memory may also play a role in the detection of the potential distraction: activity in right prefrontal cortex was greater in participants who experienced relatively strong attentional capture, but only when working memory load was high and the distractor singleton present.

Other findings also support the notion that working memory load affects the extent to which distractors are processed, even when these are not directly associated with a task response. The Ebbinghaus (or Titchener) illusion is a visual size illusion in which the perceived size of a target circle is affected by the size of surrounding inducer circles, such that a target surrounded by large inducers has a smaller perceived size compared to a target surrounded by small inducers (e.g., Roberts et al., 2005). The magnitude of the Ebbinghaus illusion can be seen as an index of selective attention, in that greater processing of the task-irrelevant inducers should lead to more illusion. Indeed, individuals who show superior selective attention on a range of tasks also experience very little Ebbinghaus illusion (De Fockert et al., 2011; Caparos et al., 2012, 2013). When the Ebbinghaus illusion is measured during performance of a concurrent working memory task, observers experience more Ebbinghaus illusion when they are maintaining a large digit set in working memory, compared to a small set (De Fockert and Wu, 2009). The same manipulation of working memory load has also been shown to affect inattentional blindness. When observers perform a demanding perceptual task involving comparing the sizes of two centrally presented lines, the one-off occurrence of an unexpected additional visual stimulus often goes unnoticed (Rock et al., 1992; Mack and Rock, 1998). Such inattentional blindness, however, can be eliminated by high working memory load (De Fockert and Bremner, 2011), suggesting that the task-irrelevant critical stimulus is more likely to be perceived when working memory is unavailable to maintain attention on the relevant task. A similar effect of working memory load on the processing of an irrelevant stimulus that is entirely task-irrelevant was shown by Carmel et al. (2012), who found that an irrelevant face presented alongside a relevant name categorization task was more likely to be subsequently identified when concurrent working memory load was high. Interestingly, this effect of working memory load was not found when the distracting images were buildings, suggesting that ignoring distractors that are salient (like faces) may be especially reliant on the availability of working memory.

The evidence that working memory is involved in preventing processing for distractors that are not directly associated with any task-relevant response is important, as it suggests that the effect of working memory on selective attention occurs at the level of sensory processing, rather than response selection. The effects of working memory load on attentional capture, the Ebbinghaus illusion, and detection of the critical item in inattentional blindness are unlikely to reflect a greater tendency to activate an incorrect response code following processing of the distractor. The attentional capture task requires responding to the orientation (horizontal, vertical) of a line in a target shape, and the non-target shapes never contain horizontal or vertical lines. Instead, the distracting singleton is defined by its unique color, which is not a relevant dimension in the task. In the Ebbinghaus task, response profiles would be opposite to those observed if people would mistakenly respond to the size of the distractors. In the inattentional blindness task, the critical item occurs only once and is not associated with any task response. It is thus unlikely that working memory acts on selective attention by preventing activation of responses associated with the distractors. Instead, the increases under high working memory load of the attentional capture effect, the Ebbinghaus illusion, and detection rates in inattentional blindness, seem to result from greater perception of the distractors under high load. This conclusion is further supported by fMRI evidence that the effects of working memory on distractor processing can be seen as early as V1 in primary visual cortex (Kelley and Lavie, 2011).

In addition to studies showing that working memory load leads to greater distractor interference when stimuli for both tasks are presented visually, there is also evidence that distractor processing increases when the working memory and selective attention tasks involve different sensory modalities (Dalton et al., 2009a,b). High load on a working memory task involving sub-vocal rehearsal of visually presented digits, during a task that required attending to target sounds and ignoring distractor sounds, led to greater interference from the auditory distractors (Dalton et al., 2009a). The same effect of increasing distractor interference when maintaining a large visually presented digit set in working memory has been shown when attention has to be directed toward a tactile target while ignoring an irrelevant tactile distractor (Dalton et al., 2009b). Conversely however, the difference in cost on the processing of visual targets produced by either tactile or painful distractors is reduced following a moderate increase in visual working memory load (Legrain et al., 2011), a result that could be interpreted to show that working memory led to a reduction in the attention capturing effect of the painful distractors. This suggests that working memory is more involved in reducing interference from distractors in the same modality as the target, compared to from those in a modality other than the target modality. Further work is required to test this speculation, directly comparing interference from same (vs. different) modality distractors on target processing under varying levels of load.

Although there is now evidence from a range of experimental tasks and measures that the unavailability of working memory during selective attention can increase the processing of distractors, the growing body of work on the role of working memory in selective attention has also occasionally found that working memory load can have different effects on distactibility. High working memory load has repeatedly been shown to either increase or reduce distractibility, depending on whether the contents of the working memory task overlap with the processing of the target or the distractor in a selective attention task, respectively (Kim et al., 2005; Park et al., 2007; de Liaño et al., 2010). High working memory load involving maintaining a set of letters leads to greater processing of the irrelevant color of a Stroop color word when the meaning of the word has to be attended (and the color ignored), but to reduced processing of the irrelevant word when the color has to be attended (and word meaning ignored). High load on a working memory task for spatial location has no effect on distractor processing in either case (Kim et al., 2005; de Liaño et al., 2010). Similarly, when the working memory task involves memorizing either faces or houses, and the selective attention task also requires attending to faces and ignoring houses, or vice versa, distractor effects are increased when the working memory items are of the same category as the targets in the attention task, but reduced when they are the same as the distractors (Park et al., 2007). These findings imply that working memory can be loaded for a specific stimulus category, and that the loaded category has to overlap with target processing in order to lead to an increase in distractor processing. Other work also suggests that cognitive load can have opposite effects on distractibility depending on the nature of the distracting information. Boot et al. (2005) found that, whereas attentional capture by color singletons was increased under high cognitive load, capture by sudden onsets was reduced. As argued below, the salience of the distractor may be an important factor determining whether working memory load will affect distractor processing.

How does the notion that the contents of the working memory load need to overlap with target processing (Kim et al., 2005; Park et al., 2007) correspond with other evidence? In many studies showing an increase in distractibility under high working memory load, the load manipulation involved maintaining digit sets or digit order. This working memory task indeed sometimes overlapped with target processing on the selective attention task. When the selective attention task required attending to target names and ignoring distractor faces (De Fockert et al., 2001), the working memory task can be argued to have more in common with the verbal requirements of classifying the target names than with identifying the distractor faces. Other findings, however, do not correspond well with the claim that the type of working memory load has to show more overlap with target than with distractor processing in order to increase distractibility. Greater distractor processing has been observed when the working memory task overlaps equally with target and distractor processing, such as when both target and distractor are letters (Lavie et al., 2004) or arrows (Pratt et al., 2011). Moreover, working memory load for digits also increases distractor processing when the target task requires size judgments (De Fockert and Wu, 2009; De Fockert and Bremner, 2011), and even when the selective attention task involves a different modality to the working memory task (Dalton et al., 2009a,b). It seems that working memory load is capable of increasing distractibility even if the overlap between the contents of the working memory task and target processing in selective attention is minimal.

In our own work, we have also come across cases in which high working memory load did not produce an increase in the level of distractor processing. In a recent fMRI study, we found that the behavioral attentional capture effect was not enhanced by high working memory load, even though the response in inferior frontal gyrus showed a reliable interaction between working memory load and the presence of the distracting singleton (De Fockert and Theeuwes, 2012). In that study, conditions in which the color singleton was present or absent were blocked. It is possible that this reduced the salience of the distractors, as their presence was perfectly predictable, unlike in previous work that did show an effect of working memory load on attentional capture by color singletons, but in which trials on which the distractor singleton was present and absent either occurred randomly within a block (Lavie and De Fockert, 2005), or in which the singleton color could coincide with either the target or a distractor within a block (Boot et al., 2005). Other work has also shown that the processing of expected distractors is less likely to be affected by working memory load (Macdonald and Lavie, 2008). Additionally, as shown by Carmel et al. (2012), distractor processing is only affected by working memory load when the distractor is sufficiently salient (faces, rather than buildings in their study), although the influence of visual working memory load on Simon interference effects (produced by the irrelevant location of a target stimulus, arguably a particularly salient form of interference) is less clear (Stins et al., 2004), and emotional faces produce interference regardless of the level of concurrent working memory load (Pecchinenda and Heil, 2007, Experiment 3). Together, these findings suggest that distractor salience is an important factor affecting whether working memory load influences selective attention. Effects of working memory load on interference may show an inverted-U shaped function as distractor salience increases: non-salient distractors (e.g., buildings) can be ignored even when working memory is loaded, whereas highly salient distractors (e.g., emotional faces, the irrelevant target location in the Simon task, sudden onset singletons) are processed even when working memory is available. When the salience of the distractor falls between these extremes (e.g., faces, letters associated with a task response, color singletons), working memory is able to prevent distractor processing. Further work is needed to systematically investigate the role of distractor salience.

Distraction by deviant sounds has also been found to be reduced under high working memory load (Berti and Schröger, 2003; SanMiguel et al., 2008), although the increase in working memory load (from *n* = 0 to *n* = 1 on an *n*-back task) may have insufficiently loaded working memory to lead to an increase in distractibility in these studies. In another recent study, we found that high working memory load can lead to significantly reduced distractibility in the Navon task (Ahmed and De Fockert, 2012a). In the Navon task, global stimuli consist of many local elements, and attention has to be directed to either the global shape or the local elements (Navon, 1977). Distractibility can be measured by comparing performance in conditions in which information at the attended and unattended levels is compatible (vs. incompatible). When attention was directed to the local level, working memory load had the expected effect of increasing distractibility from the global level. Conversely however, distractor effects were reduced by high working memory load when attention was directed to the global level, and the local level had to be ignored. Below, we will discuss these findings further when we consider a possible mechanism of the effect of working memory load on attention.

To sum up the work using manipulations of working memory load during selective attention, there is much evidence that processing of task-irrelevant information is enhanced when load on a concurrent task of working memory is high, implying that working memory plays a role in the active control against distractor interference. The effect of working memory on selective attention has been demonstrated in Stroop-type tasks, where the distractor is associated with one of the task responses, but also in tasks in which the distractor cannot lead to response activation, suggesting that the effect of working memory has an early locus in attention. In contrast, there are also examples in which high working load has not led to an increase in the extent of distractor processing in selective attention. These include situations in which the content of the working memory task overlaps mostly with the processing of the distractor (rather than the target) in selective attention (e.g., Kim et al., 2005), although there are many studies in which high working memory load does lead to greater distractor processing despite minimal overlaps between the working memory task and target processing. Distractor processing may be more likely to increase under high working memory load when distractors are likely to cause interference, either because they are associated with a task-relevant response, or because they have an intermediate level of salience or occur unpredictably from trial to trial.

On balance, the evidence for greater distractor processing under high working memory load seems stronger than the evidence for the opposite effect. Indeed, a meta-analysis on the effect sizes reported in 26 studies (49 experiments) manipulating working memory load during selective attention shows that the prevailing effect of working memory load is to increase distractor processing [mean *r* = 0.202, t_(48)_ = 2.95, SEM = 0.0686, *p* < 0.005; see Table 1]. This is a strong finding, as it includes experiments that found reversed effects (reduced distractibility under load) that were nevertheless predicted on the basis of changes to the spatial profile of attention, and that also showed that high working memory load did increase distractor processing when expected to do so (see below; Ahmed and De Fockert, 2012a,b). In addition, there were significantly more demonstrations of increases in distractibility under high working memory load (35 experiments) than reductions [14 experiments; χ^2^_(1)_ = 9.0, *p* < 0.01, two-tailed], although the magnitude of the effect was the same in studies showing an increase (mean *r* = 0.477) and those showing a reduction (mean *r* = 0.484, *p* > 0.9). We note that any bias against publication of null results means that unreported failures to replicate the original effect are likely to exist, although such bias will not have affected the number of reported reliable reversals of the effect.

**Table 1:** **Effect sizes (*r*) for the effect of working memory (WM) load (unless stated otherwise) on distractor processing (reaction time effects, unless stated otherwise)**.

**Source**	**Experiment**	**Effect size (*r*)**	**Effect of working memory load on**
De Fockert et al. (2001)		**0.773**	Interference from distractor faces
Berti and Schröger (2003)		**−0.644**	Distraction by auditory deviant
Lavie et al. (2004)	1	**0.604**	Interference from distractor letters (flanker interference)
Lavie et al. (2004)	2	**0.514**	Flanker interference with articulatory suppression
Lavie et al. (2004)	3	**0.667**	Flanker interference under low, high perceptual load
Lavie et al. (2004)	4	**0.752**	Flanker interference (single vs. dual task with high load)
Lavie et al. (2004)	5	**0.836**	Flanker interference (single vs. dual task with low load)
Stins et al. (2004)	1	**0.369**	Stroop interference (spatial WM task)
Stins et al. (2004)	2	**−0.440**	Simon congruence effect (spatial WM task)
Boot et al. (2005)	1	0.410	Attentional capture by onset singletons
Boot et al. (2005)	2	**0.414**	Attentional capture by color singletons
Lavie and De Fockert (2005)	1	**0.653**	Attentional capture by color singletons
Lavie and De Fockert (2005)	2	**0.642**	Attentional capture by color singletons
Kim et al. (2005)	1a	**0.658**	Stroop interference (verbal WM condition, target load)
Kim et al. (2005)	2a	**−0.638**	Stroop interference (verbal WM condition, distractor load)
Kim et al. (2005)	3a	**0.660**	L/R congruency (verbal WM condition, target load)
Kim et al. (2005)	3b	−0.548	L/R congruency (verbal WM condition, distractor load)
Park et al. (2007)	1	**0.335**	Interference on same/different judgments (target load)
Park et al. (2007)	1	**−0.299**	Interference on same/different judgments (distractor load)
Park et al. (2007)	2	**0.464**	Interference on same/different judgments (target load)
Park et al. (2007)	2	**−0.483**	Interference on same/different judgments (distractor load)
Chen and Chan (2007)	3	0.052	Flanker interference (narrow focus condition)
Pecchinenda and Heil (2007)	1	**0.447**	Interference from distractor faces
Pecchinenda and Heil (2007)	2	**0.426**	Interference from distractor faces
Pecchinenda and Heil (2007)	3	-0.062	Interference from emotional distractor faces
SanMiguel et al. (2008)		**−0.703**	Distraction by auditory deviant
Macdonald and Lavie (2008)	6	0.336	Detection of expected stimulus during letter search
Dalton et al. (2009a)		**0.443**	Interference from auditory distractors
Dalton et al. (2009b)	1	**0.505**	Interference from tactile distractors (accuracy rates)
Dalton et al. (2009b)	2	**0.455**	Interference from tactile distractors (accuracy rates)
De Fockert and Wu (2009)		**0.660**	Ebbinghaus illusion
Kelley and Lavie (2011)		**0.360**	Interference from distractor objects
de Liaño et al. (2010)	1	**−0.421**	Stroop interference (distractor load) (inverse efficiency scores)
De Fockert et al. (2010)	2	**0.453**	Flanker interference (prime display)
Jongen and Jonkman (2011)		0.013	Interference from distractor faces
Legrain et al. (2011)		**−0.768**	Capture by painful (vs. non-painful) tactile distractors
Pratt et al. (2011)		**0.567**	Interference from distractor arrows (accuracy rates)
De Fockert and Bremner (2011)	1	**0.483**	Target detection in inattentional blindness
De Fockert and Bremner (2011)	2	**0.421**	Target detection in inattentional blindness
De Fockert and Theeuwes (2012)		-0.465	Attentional capture by color singletons
Carmel et al. (2012)	1	**0.225**	Distractor face identification
Carmel et al. (2012)	2	**0.327**	Distractor face identification
Carmel et al. (2012)	3	0.096	Distractor house identification
Ahmed and De Fockert (2012a)	1	**0.613**	Navon interference from global level
Ahmed and De Fockert (2012a)	2	**−0.620**	Navon interference from local level
Ahmed and De Fockert (2012a)	3	**0.291**	Navon interference from global level
Ahmed and De Fockert (2012a)	3	**−0.261**	Navon interference from local level
Ahmed and De Fockert (2012b)	1	**0.763**	Flanker interference (High WM capacity)
Ahmed and De Fockert (2012b)	1	−0.422	Flanker interference (Low WM capacity)

Positive effect sizes represent cases where distractor processing was greater under high (vs. low) working memory load. Negative effect sizes represent cases where distractor processing was greater under low (vs. high) working memory load. Effect sizes in bold are statistically significant effects at p < 0.05. Papers included in the meta-analysis were first identified via PubMed (search terms “working memory selective attention”). The search returned 750 articles, from which relevant papers were selected, i.e., when they measured distractor processing in selective attention whilst manipulating working memory load. In addition, any relevant work was included that was cited in the selected papers, but had not been identified in the PubMed search.

The finding that high working memory load is associated with increased distractor processing is in line with load theory of selective attention (Lavie et al., 2004). According to the model, active control against processing of perceived distractors requires the availability of working memory, in order to maintain a clear distinction between relevant and irrelevant processing. High load on a working memory task that has to be performed concurrently with a selective attention leaves less capacity available for the prioritization of relevant targets, leading to the greater distractibility found in the studies reviewed here.

## Individual differences in working memory capacity and distractibility

The evidence discussed so far for a role of working memory in distractor processing has been based on manipulations of working memory load, usually within the same participants. A converging line of evidence for the claim that working memory is involved in the extent of distractor processing in selection comes from studies investigating selective attention in individuals with varying levels of working memory capacity. The rationale is that the availability of working memory for selective attention is chronically reduced in individuals with low working memory capacity, compared to those with higher capacity. Consequently, differences in distractibility between individuals with either low or high working memory capacity should be similar to differences in distractibility within the same the person under either high working or low working memory load, respectively.

There is much evidence that attention performance is associated with working memory capacity, often showing that individuals with greater working memory capacity are more efficient at focusing their attention to task-appropriate information (the “controlled attention theory of working memory,” e.g., Engle et al., 1999; Conway et al., 2001; Kane and Engle, 2003; Engle and Kane, 2004). For example, when repeating an attended auditory message while ignoring a simultaneously presented irrelevant message, low working memory span individuals are three times more likely than high span individuals to report hearing their own name in the irrelevant message (Conway et al., 2001). High working memory span individuals are also faster than those with lower span at identifying a target letter at an uncued location, implying better attentional control in the high span individuals (Kane et al., 2001). Performance in high span individuals, compared to low span individuals, is less affected by the presence of an unexpected auditory deviant and their ERPs also show a smaller N1 component associated with infrequent auditory stimuli, again suggesting they are better at preventing processing for the irrelevant distractors (Sörqvist et al., 2012; Tsuchida et al., 2012). Much indirect evidence for an association between working memory capacity and the level of interference produced by irrelevant distractors has come from studies on cognitive ageing, which tend to show that elderly participants are disproportionally impaired compared to younger participants at tasks that require active rejection of distracting information (Baddeley, 1996; De Fockert, 2005; De Fockert et al., 2009). Such evidence for a link between working memory capacity and selective attention is mostly indirect, as a reduction in working memory capacity in the older (vs. younger) groups is often assumed (e.g., Welford, 1958) rather than measured in these studies.

A number of studies have investigated the link between working memory capacity and distractor processing in selective attention more directly, by taking measures of working memory span in young participants, and comparing selective attention performance between groups of low and high span. A measure of a person's working memory capacity is typically obtained with a standard span task such as the Operation Span (Ospan) task (Unsworth et al., 2005). In the Ospan, participants perform number calculations while adding to a list of words they keep in memory, and working memory span is the sum of all correctly recalled word lists. In a standard Stroop task requiring naming of the ink color of printed letter strings (Stroop, 1935), interference effects produced when the letter string reads another color name are consistently greater in individuals with low, compared to those with high working memory span (Kane and Engle, 2003). Greater interference effects also occur in Eriksen-type flanker tasks in low (vs. high) span individuals (Ahmed and De Fockert, 2012b; Shipstead et al., 2012). Together, these findings suggest that having low working memory capacity has the same effect on distractibility as having a high load on working memory.

A few studies have failed to find clear evidence that low working memory span is always associated with reduced attentional selectivity. In a recent study, only one third of self-reported everyday failures of attention showed a significant correlation with working memory span (Unsworth et al., 2012), although the reported attention failures mostly involved absent-mindedness and mind wandering, rather than measures of selective attention. Another recent study found that the higher distractibility in low (vs. high) span individuals can be reversed when working memory load is manipulated at the same time (Ahmed and De Fockert, 2012b). Whereas flanker interference effects were greater in low (vs. high) span individuals when load on a concurrent working memory task was low, interference effects showed the opposite pattern under high working memory load, so that low span individuals became less distracted than those with high span. We return to this finding in the next section when we discuss possible mechanisms by which working memory may affect selective attention.

In sum, the notion that working memory plays a role in selective attention is well-supported by studies on individual differences in working memory. Perhaps more so than within-participant manipulations of working memory availability, which have produced some conflicting results, individual differences in working memory capacity are fairly consistently found to be associated with differences in selective attention, such that low working memory span is associated with reduced performance on selective attention tasks, including tasks involving ignoring potentially distracting information. A meta-analysis on the effect sizes reported in studies manipulating measuring selective attention as a function of working memory capacity shows that high working memory capacity is associated with reduced distractor interference [mean *r* = 0.286, t_(11)_ = 7.92, SEM = 0.0361, *p* < 0.001; see Table 2]. This finding is in line with load theory of selective attention (Lavie et al., 2004), which predicts that any reduction in the availability of working memory, be it because of high concurrent load on working memory, as discussed in the previous section, or low working memory capacity, as outlined in the current section, will compromise the ability to effectively control against processing of perceived distractors.

**Table 2:** **Effect sizes (*r*) for the effect of working memory capacity on distractor processing (reaction time effects, unless stated otherwise; high vs. low score on working memory span measure, unless stated otherwise)**.

**Source**	**Experiment**	**Effect size (*r*)**	**Effect of working memory capacity on**
Conway et al. (2001)		**0.416**	Shadowing cost during presentation of irrelevant own name
Kane and Engle (2003)	1	**0.289**	Stroop interference (error rate)
Kane and Engle (2003)	2	**0.232**	Stroop interference with feedback (error rate)
Kane and Engle (2003)	3	**0.295**	Stroop interference
Kane and Engle (2003)	4	**0.218**	Stroop interference
De Fockert et al. (2009)		**0.535**	Interference from irrelevant faces (young vs. old participants)
Poole and Kane (2009)	1	**0.217**	Visual search in the presence of distractors
Poole and Kane (2009)	2	**0.323**	Visual search in the presence of distractors
Poole and Kane (2009)	3	**0.246**	Visual search in the presence of distractors
Shipstead et al. (2012)		**0.367**	Flanker interference in displays without placeholders
Shipstead et al. (2012)		0.020	Flanker interference in displays with placeholders
Sörqvist et al. (2012)		**0.271**	Effect of auditory deviant on target processing

In all cases, distractor processing was greater in participants with low (vs. high) working memory capacity. Effect sizes in bold are statistically significant effects at p < 0.05. Papers included in the meta-analysis were first identified via PubMed (search terms “working memory selective attention”). The search returned 750 articles, from which relevant papers were selected, i.e., when they measured distractor processing in selective attention as a function of working memory capacity. In addition, any relevant work was included that was cited in the selected papers, but had not been identified in the PubMed search.

## Possible mechanisms underlying the link between working memory and selective attention

Although the extant evidence clearly suggests a degree of functional overlap between working memory and selective attention, until recently the exact nature of the interaction between working memory and selective attention remained relatively underspecified. The relationship between working memory and selective attention may simply involve relying on the same limited resource pool needed both for active information maintenance in working memory and for active distractor suppression in selective attention. Alternatively, working memory may play a more specific role in selective attention, for example by maintaining clear priorities for target-related processing in selective attention (Lavie et al., 2004). Two issues will be discussed in this section. Working memory is a multi-component system, and the first question concerns whether any specific working memory component(s) are involved in selective attention, and if so, what these components might be. Second, whereas the effect of working memory on selective attention is well-documented in terms of the mere extent to which distracting information is processed, it remains unclear which functional mechanism in selective attention underlies the effect.

Which working memory component(s) may be involved in selective attention? Many studies on the role of working memory in selective attention have used verbal working memory tasks to manipulate load (e.g., Lavie et al., 2004). The finding that verbal working memory load leads to an increase in distractor processing in selective attention may point at the phonological loop of the working memory system as the key subsystem, especially since a non-verbal working memory task such as working memory for spatial locations does not always have the same effect of increasing distractibility in selective attention (Kim et al., 2005; but see Stins et al., 2004, for evidence that high load on spatial working memory leads to more Stroop interference). Two findings, however, argue against this conclusion. First, the effect of working memory load on distractibility persists even when the phonological loop is loaded by overt rehearsal in both high and low load (Lavie et al., 2004, Experiment 2). Second, manipulations of cognitive control load other than verbal working memory also lead to increased distractibility (Lavie et al., 2004; De Fockert et al., 2010). Distractor effects are greater when there is a cost of concurrence (Navon and Gopher, 1979), such as when the task involves switching between a working memory and a selective attention task, compared to when the selective attention task is performed on its own (Lavie et al., 2004, Experiments 4 and 5). Similarly, occasionally having to make a spatially incongruent response, whilst withholding the spatially prepotent congruent response that is required on a majority of trials (a manipulation that does not load working memory), also leads to increased distractibility on a subsequent flanker task (De Fockert et al., 2010).

The findings that manipulations that involve cognitive control functions other than working memory, such as dual task performance and response inhibition, are also associated with an increase in distractor processing in selective attention, suggest that the effect of working memory in selective attention is more likely to be domain-general than domain-specific (De Fockert and Bremner, 2011). The domain-general nature of the role of working memory in selective attention is further demonstrated by the finding that verbal working memory load affects the processing of irrelevant information in tasks that are unlikely to rely on activation of verbal codes, such as visual search in the attentional capture studies (Lavie and De Fockert, 2005), and size judgments in the Ebbinghaus illusion (De Fockert and Wu, 2009) and inattentional blindness (De Fockert and Bremner, 2011). An obvious candidate for a domain-general component of working memory that is involved in selective attention is the central executive. Indeed, one of the original functions of the central executive was proposed to be selective attention to task-relevant information in the face of potential distraction by other sources (Baddeley, 1996). The central executive, however, has no direct storage role, and storage is often what is manipulated to increase load on working memory. Together with the finding that manipulations loading cognitive control functions other than working memory, including dual task performance and suppression of prepotent responses, have also been shown to increase distractor processing in selective attention, this leaves open the possibility that working memory has an indirect effect on selective attention, perhaps because it shares limited resources with cognitive control mechanisms involved in active distractor rejection. More work is needed to further explore this possibility.

Which functional mechanisms in selective attention may explain the observed variations in distractibility as a function of the availability of working memory? At least two possibilities have been suggested, based on either temporal or spatial aspects of selective attention. High load on working memory leads to a delay in the allocation of attention to target representations in visual processing (Scalf et al., 2011): the neural response in occipital cortex associated with a visual target peaked later when load on a concurrent working memory task was high (vs. low), suggesting that visual attention is deployed more slowly to the relevant target representation when working memory is otherwise engaged. Other findings support the notion that working memory availability affects the timing of attentional processes (e.g., Heitz and Engle, 2007; Poole and Kane, 2009). During the processing of flanker trials, individuals with low and high working memory capacity are initially equally distractible by irrelevant information, as they show similar accuracy for responses faster than ~400 ms. Performance improves more rapidly however in high (vs. low) span individuals as responses get slower, which could suggest that the high span individuals are quicker to constrain attention to the relevant information (Heitz and Engle, 2007).

Working memory may also affect the way in which selective attention operates in the spatial domain. The evidence that high individuals are faster than those with a low working memory span to constrain spatial attention to task-relevant locations, thereby excluding distractors locations from processing (Heitz and Engle, 2007; Poole and Kane, 2009), suggests that both temporal and spatial aspects of selective attention are affected by working memory. High working memory capacity has also shown to allow better constraining of spatial attention over a prolonged time period (Poole and Kane, 2009). Further evidence for the notion that working memory load affects the distribution of spatial attention comes from a study using a flanker task in which distractor interference was measured at varying spatial separations between the target and the distractor under low or high working memory loads (Caparos and Linnell, 2010). High load had the effect of dispersing the characteristic spatial profile of attention in terms of facilitation and suppression zones (Müller et al., 2005). Similar findings have been reported in a flanker task while manipulating the factors of working memory load and working memory capacity simultaneously (Ahmed and De Fockert, 2012b). The spatial profile of attention was most constrained in high working memory span individuals while load on the working memory task was low. Similar, and more dispersed profiles than in the high span group under low load were observed under high load in the high span group, and under low load in the low span group. When working memory was high in the low span group, spatial attention became even less constrained. These findings all suggest that working memory is necessary to maintain a task-appropriate narrow attentional focus, and that the unavailability of working memory leads to a widening of the attentional focus. Indeed, effects of working memory load on distractibility are absent when attentional focus is experimentally manipulated to remain either narrow or wide (Chen and Chan, 2007).

The combined effect of working memory load and capacity on the spatial profile of attention can explain the seemingly contradictory findings that working memory load can have opposite effects depending on a person's working memory capacity, increasing distractibility in individuals with high working memory span, but reducing distractibility in low span individuals (Ahmed and De Fockert, 2012b). The spatial profile of attention consists of alternating facilitation and suppression zones (Müller et al., 2005), and the pattern of distractor effects as a function of both working memory load and capacity is accurately explained in terms of the region of the attentional profile (facilitation, suppression) that the distractor coincides with, giving the presumed changes in the spatial dispersion of the attentional profile as a function of working memory load and capacity. Similar changes in the spatial profile of attention may also explain the finding that interference effects from the global level of a Navon figure are increased, but interference effects from the local level are reduced by high working memory load (Ahmed and De Fockert, 2012a). A more dispersed attentional profile under high load will increase the likelihood that the global level of the Navon figure is attended, leading to greater interference when attention has to be directed toward the local level, but less interference when it has to be directed toward the global level.

In summary, the effect of working memory on selective attention is largely domain-general, and working memory and selective attention interact even when the tasks show little overlap in terms of stimulus content (but see Kim et al., 2005; Park et al., 2007, for findings suggesting domain-specific effects of working memory on selection). Moreover, there are at least two possible ways in which selective attention can be influenced by working memory. First, the temporal dynamics of attentional selection may change when working memory is unavailable for selection. Under high working memory load, activation of the representation of a visual target is delayed, leaving more opportunity for distractors to influence behavior. Similarly, low working memory capacity is associated with slower constraining of attention to task-relevant inputs, again leaving a longer time window in which distractors may be processed. Second, the spatial distribution of attention varies as a function of the availability of working memory. Spatial attention has a more constrained focus when working memory is available for attention (under low working memory load, or in individuals with high working memory span), compared to when working memory is less available (under high working memory load, or in individuals with low working memory span).

## Discussion

The idea that working memory and selective attention are closely related systems has become mainstream during the past decade, to the extent that they are sometimes seen as two manifestations of the same underlying system (Awh and Jonides, 2001; Awh et al., 2006; Chun, 2011). Both working memory and selective attention involve prioritization of certain information in the presence of competing inputs, and involve maintaining information across time (working memory) and space (selective attention). Furthermore, working memory and selective attention share neural systems (e.g., Gazzaley et al., 2007; Mayer et al., 2007; Gazzaley and Nobre, 2012), and people's performance on working memory and selective attention tasks shows a consistent positive correlation (e.g., Kane and Engle, 2003). The work reviewed here has found with reasonable consistency that the unavailability of working memory for selective attention, either because working memory is engaged in an additional task of high load or because there is low working memory capacity, is associated with greater distractibility in a range of selective attention tasks. This conclusion is supported by a meta-analysis of the effect sizes in studies manipulating working memory load during selective attention, which found that high working memory load tends to lead to an increase in distractor processing.

An alternative account for the observed effects of working memory on selective attention is that the increase in distractibility under high working memory load is merely due to the increase in overall task difficulty when working memory load is high. Two lines of evidence argue against this argument. First, when there is an increase in overall response latency and/or error rate under high working memory load, suggesting a general increase in task difficulty, the increase in the distractor interference effect tends to be disproportionate to the increase in the overall performance cost (e.g., Lavie et al., 2004). Second, performance that benefits from greater distractor processing should be better under high working memory load. There are a few examples that this is indeed the case. Negative priming refers to the finding that processing is impaired for recently ignored information (Tipper, 1985). Therefore, if distractors cannot be efficiently ignored under high working memory load, then negative priming effects should be reduced by high load. This is indeed the case, and the performance impairment normally observed when a previously ignored distractor is repeated as a target is eliminated by high working memory load (De Fockert et al., 2010). Another example in which performance was improved by high load on working memory is the release from inattentional blindness found when detection and identification rates for an unexpected task-irrelevant visual stimulus were measured under varying levels of working memory load (De Fockert and Bremner, 2011).

Load theory predicts opposite effects on distractibility as a function of increases in perceptual load and working memory load (Lavie et al., 2004). However, whether the constructs of perceptual load and working memory load can be clearly distinguished has been questioned, and it has been suggested that the prediction of increased distractibility under high working memory load only holds if perceptual and working memory load are independent and rely on separate resources (Tsal and Benoni, 2010). If they do not, then any additional increase in the load on attentional resources (either perceptual or cognitive) should be associated with reduced distractor processing. The evidence from studies on working memory load and working memory capacity clearly does not support this interpretation of load theory, suggesting that cognitive and perceptual load have to be regarded as separate constructs. Indeed, whereas spatial working memory and spatial selective attention may share certain resources (Awh and Jonides, 2001), there is less evidence for any direct resource competition between the types of working memory discussed here (e.g., working memory for digits) and visual selection. For example, non-overlapping cortical regions are involved in tasks of working memory for digit order and visual selection (e.g., De Fockert et al., 2001). In addition, perceptual load and working memory load are repeatedly shown to have opposite effects on selection in studies on either perceptual or working memory load, and also when both types of load are manipulated within the same experiment, distractor processing is reduced when perceptual load is high, but increased when working memory load is high (Lavie et al., 2004, Experiment 3). Other work has found a similar dissociation between the effects of the two types of load on distractor processing (Yi et al., 2004).

A number of key questions about the role of working memory in selective attention are now beginning to be addressed. First, it seems that working memory influences selection at an early stage of processing, often affecting the perception of distracting information (e.g., De Fockert and Bremner, 2011). Second, the effect of working memory on selective attention is domain-general, as loading working memory in one domain (e.g., maintaining visually presented digits) can lead to an increase in distractor processing in a different domain (e.g., processing of a color singleton or distracting circle; e.g., Lavie and De Fockert, 2005; De Fockert and Wu, 2009) or even in a different modality (e.g., audition or touch; Dalton et al., 2009a,b). Moreover, other manipulations of cognitive control (e.g., dual task performance and response suppression) affect distractor processing in a similar way to loading working memory. Third, loading working memory can affect both the temporal and the spatial deployment of attention (Caparos and Linnell, 2010; Scalf et al., 2011; Ahmed and De Fockert, 2012b).

To conclude, this paper has provided a review of the evidence for a form of attentional selection that is different from the type of selective processing, based on perceptual aspects of the input, proposed by the perceptual load and dilution models. Whereas selection can often occur passively because of the characteristics of the input (i.e., under conditions of high perceptual load or high dilution), distracting information often receives some processing and needs to be actively selected against, for example when distractors are sufficiently salient, or unexpected. In such cases of potential distraction, working memory plays a role in minimizing the interference produced by the distractors. This is but one of the ways in which working memory and selective attention are likely to interact, and other links include the role of selective attention in determining which information is encoded in working memory (e.g., Oberauer, 2003), and the finding that the contents of working memory can bias what is selected in visual processing (Soto et al., 2008). These multiple interactions emphasize the close relationship between working memory and selective attention.

### Conflict of interest statement

The author declares that the research was conducted in the absence of any commercial or financial relationships that could be construed as a potential conflict of interest.
